# Synthetic Cannabinoids in Prisons: Content Analysis of TikToks

**DOI:** 10.2196/37632

**Published:** 2022-05-31

**Authors:** Tiana J McMann, Alec Calac, Matthew Nali, Raphael Cuomo, James Maroulis, Tim K Mackey

**Affiliations:** 1 Global Health Program Department of Anthropology University of California San Diego La Jolla, CA United States; 2 Global Health Policy and Data Institute San Diego, CA United States; 3 S-3 Research San Diego, CA United States; 4 Department of Anesthesiology University of California San Diego San Diego, CA United States

**Keywords:** social media, substance use disorder, synthetic drugs, prison, cannabinoid, synthetic, psychoactive, illicit, video, substance use, harmful, K2/Spice, TikTok

## Abstract

**Background:**

Synthetic cannabinoids are a significant public health concern, especially among incarcerated populations due to increased reports of abuse. Recent news reports have highlighted the severe consequences of K2/Spice, a synthetic cannabinoid, among the prison population in the United States. Despite regulations against cell phone use, inmates use TikTok to post K2/Spice-related content.

**Objective:**

This study aimed to examine TikTok posts for use and illicit distribution of psychoactive substances (eg, K2/Spice) among incarcerated populations.

**Methods:**

The study collected TikTok videos associated with the #k2spice hashtag and used a data collection approach similar to snowball sampling. Inductive coding was used to conduct content analysis of video characteristics. Videos were manually annotated to generate binary classifications related to the use of K2/Spice as well as selling and buying activities associated with it. Statistical analysis was used to determine associations between a video’s user engagement and an intent to buy or sell K2/Spice.

**Results:**

A total of 89 TikTok videos with the hashtag #k2spice were manually coded, with 40% (n=36) identified as displaying the use, solicitation, or adverse effects of K2/Spice among the prison population. Of them, 44.44% (n=16) were in a prison-based setting documenting adverse effects including possible overdose. Videos with higher user engagement were positively correlated with comments indicating an intent to buy or sell K2/Spice.

**Conclusions:**

K2/Spice is a drug subject to abuse among prison inmates in the United States, including depictions of its harmful effects being recorded and shared on TikTok. Lack of policy enforcement on TikTok and the need for better access to treatment services within the prison system may be exacerbating substance use among this highly vulnerable population. Minimizing the potential individual harm of this content on the incarcerated population should be a priority for social media platforms and the criminal justice system alike.

## Introduction

Synthetic cannabinoids are a significant public health concern. Their use can cause several adverse effects (eg, anxiety, paranoia, tachycardia, lightheadedness) and can lead to significant health consequences [1,2]. The types and availability of synthetic cannabinoids are on the rise and are now popular internet search terms [2,3]. Between 2009 and 2018, more than 260 unique synthetic cannabinoids were identified on the market [4]. These compounds mimic the effect of naturally occurring cannabinoids and are created in a laboratory, often with higher potency and more severe adverse effects, and introduce the risk of impairment or even death [3,5]. Synthetic cannabinoids are commonly smoked or ingested and can also be stably transported on paper letters and cards [3,4].

Although comprising less than 5% of the global population, the United States accounts for more than 1 in 5 of the world’s incarcerated population [5]. Recent news reports have highlighted increasing use of synthetic cannabinoids in correctional facilities in the United States and mass intoxications in prisons internationally [6]. In 2016, the Florida Department of Corrections estimated that more than 56 kg of synthetic cannabinoids were illegally transported into Florida correctional facilities [6]. Importantly, substance use and clinical dependence among incarcerated individuals remain higher than that in the general population [7].

It is unclear how incarcerated individuals obtain contraband substances, but it may be due to inadequate screening procedures [8]. Further, treatment for substance use within prison facilities remains insufficient to meet current needs; more than half of incarcerated individuals are indicated for treatment based on current guidelines, yet only 15% receive regular treatment [9]. Despite stringent security measures in state, federal, and private detention facilities, synthetic cannabinoids and related substance use remains a critical concern among this vulnerable population [8].

TikTok is a popular social media platform with an estimated 100 million active US users, which allows users to post short-form video content from mobile devices and is known for “viral” content that is shared and promoted among its hundreds of millions of users. Although it has also been increasingly used to obtain health-related information, especially during the COVID-19 pandemic, the quality of information on TikTok can vary widely, may not always be reliable, and can be harmful [10-12]. Harmful information found on TikTok includes sharing experiences and positive sentiment related to substance use [13-15].

Concerningly, a 2016 observational study found a significant relationship between social media and synthetic cannabinoid use among young adults [16]. Further, a separate 2016 study conducted content analysis of videos on YouTube, a popular social media platform also known for user-generated content, and found videos depicting the use and promotion of K2/Spice on the platform [17]. Social media use is also prominent among incarcerated individuals and can be a means to engage in illegal activities. Additionally, viewing substance use content on social media has been identified in this population as a trigger for relapse [18]. Building on these prior studies, research specifically examining the possible use of emerging platforms such as TikTok to promote or describe synthetic cannabinoid use or sourcing among the incarcerated population is needed.

Importantly, under the Cell Phones Contraband Act of 2010, cell phones are prohibited in federal correctional facilities; however, inmates continue to transport illegal contraband through visitors or employed personnel. As cell phones have become smaller and more sophisticated, they are capable of accessing social media platforms and other mobile apps. Today, prisoners actively record and post TikTok videos under the hashtag #PrisonTikTok, a popular content category on the platform with over 3 billion views, to share their lived experiences with incarceration including recording trending dances and showing how to prepare prison meals [19-22]. However, the use of TikTok to promote K2/Spice-related content is not well understood, though prior studies have identified content on other social media platforms not related to the incarcerated population [16,17].

As the emerging public health threat of synthetic cannabinoids continues to rise, coinciding with the use of social media among incarcerated individuals, research is needed to better understand how social media platforms are being used to possibly traffic and promote synthetic drug use. Hence, our objective was to conduct a retrospective observational infoveillance study for the purposes of exploratory analysis. We used an inductive content analysis approach to assess the use of TikTok for content related to the purported use and sourcing of synthetic cannabinoids among incarcerated populations.

## Methods

### Data Collection

For this exploratory content analysis, a seeded sample of TikTok videos with the hashtag #k2spice were retrospectively identified on June 29, 2021, using structured searches on the platform without any user login or personal search history enabled. The most relevant videos to the hashtag as determined by the TikTok search results algorithm were displayed first in order of decreasing relevance. The URLs for all the public videos returned in searches were saved, and raw video data files were downloaded using a custom script built in the Python programming language. Based on a seeded sample of 89 TikTok videos of 53 unique users/creators, we then snowball sampled for additional content from TikTok users who were coded as being associated with K2/Spice content among purported prison users. The 10 most recent posts from each of these associated TikTok user public profile pages were then collected for further analysis.

### Data Analysis

Manual annotation and content analysis of the TikTok videos were conducted by TJM, AC, and MCN. Coding was approached inductively due to the exploratory nature of the study, prior studies that have examined TikTok primarily using an inductive coding approach for TikTok videos and texts, and due to the lack of existing studies that have coded TikTok content related to synthetic drugs [14]. Authors generated a data set with binary classifications of whether the video discussed content related to adverse effects, selling, and consumption of K2/Spice. Authors also recorded user engagement (views, likes, comments, and shares), whether a post reflected sale of a K2/Spice product, if a post was associated with other user comments indicating intent to buy or sell K2/Spice, and whether the video appeared to be recorded in a prison-based setting. Three authors (TJM, AC, and MCN) coded all TikTok videos independently and achieved a high intercoder reliability (κ=0.95) for codes. For inconsistent results, authors reviewed that video’s content and metadata with other authors and conferred on the correct classification. Pearson correlation coefficient was calculated to assess the association between user engagement metrics and characteristics indicating intent to buy or sell K2/Spice as well as the medium on which K2/Spice was purportedly sold.

### Ethics Approval

An ethics exemption was not sought for this study. All information collected during this study was available in the public domain, and the study did not involve any interaction with users. Any user indefinable information is removed from study results, and results are provided in the aggregate to ensure anonymity.

## Results

A total of 89 TikTok videos with the hashtag #k2spice were retrospectively collected. The earliest video with this hashtag was uploaded on September 7, 2020, and the most recent video was uploaded on June 28, 2021. In addition to our original hashtag of interest, #k2spice, hashtags that have also been confirmed as being related to synthetic cannabinoid content in prior research (eg, touchie, black mamba) were also detected within the videos depicting synthetic marijuana use [9]. After manual annotation, 41% (n=37) of TikToks/videos reviewed were determined to be nonsignal, 40% (n=36) were confirmed to include content displaying the use, solicitation, or adverse effects of K2/Spice among the prison population, and 18% (n=16) included synthetic cannabinoid–related content among nonprison populations.

**Figure 1:**
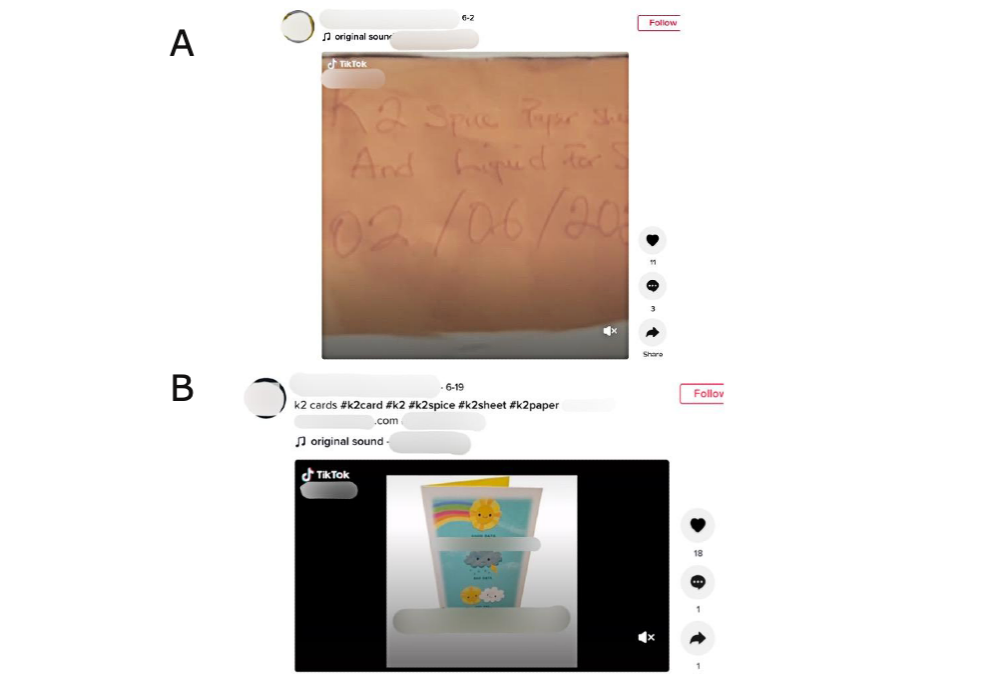
Accounts selling K2/Spice on TikTok. (A) An account showing a piece of paper advertising paper K2 and liquid K2. The paper also a date on it. (B) An account selling greeting cards with K2 on it. The video shows different greeting cards for different occasions. The K2 is inconspicuous.

Of the prison-related videos, 77.78% (n=28) were posted in 2021 and 44.44% (n=16) displayed apparent hallucinations, paranoia, aggression, heart palpitations, and nausea experienced by users portrayed in TikTok videos [1,2]. Additionally, 69.44% (n=25) of videos reviewed purported to engage in the sale of K2/Spice to other TikTok users, and 19.44% (n=7) of videos contained user comments engaged in a similar buying and selling activity (Table 1 and Figure 1 show anonymized examples). These comments were only found on prison-related videos, and 0% of comments of users purported to sell or purchase K2 were found on non–prison-related videos. Comments in response to prison-related posts revealed 2 users who disclosed the correctional facility from which they were posting, 1 medium and 1 minimum security facility, both being located in Georgia.

The contents of the 16 videos that displayed the alleged physical experiences of K2/Spice were characterized by psychomotor manifestations of inmates visibly collapsed, asleep or unconscious, staggering, and producing indecipherable noises. Videos contained a varied number of inmates, with some displaying a single person affected by K2 and others displaying upwards of 5 inmates seemingly experiencing the effects of the drug. Some videos also embedded trending audio clips typically used to generate more traffic to the content [23]. All users who posted adverse effects appeared to post secondhand experiences with synthetic cannabinoids (ie, 1 inmate recording another inmate’s alleged experience).

There was a significant positive relationship between views, comments, and shares with comments indicating an intent to buy K2/Spice (respectively, *r*=0.428, *P*=.01; *r*=0.506, *P*=.002; and *r*=0.445, *P*=.007). There was also a significant positive relationship between a video’s views, comments, and shares with comments indicating an intent to sell K2/Spice (respectively, *r*=0.470, *P*=.004; *r*=0.533, *P*=.001; and *r*=0.492, *P*=.003). There was a significant negative correlation between K2/Spice being sold via a paper medium (ie, K2/Spice allegedly sprayed on a card or paper for obfuscation and use) being shown within the video and digital comments indicating an intent to buy K2/Spice (*r*=–0.408, *P*=.02) and sell K2 spice (*r*=–0.371, *P*=.03).

**Table 1:** Examples of TikTok comments (N=561).

Theme	TikTok comments (paraphrased)	Description	Comments indicating activity, n (%)
Buy	Where can I find it? They don’t have it in my area anymore. How can I get the K2 spray online? What should I type in to order it?	An account commenting on a TikTok video, wanting to obtain K2.	9 (1.6)
Sell	Email me to place an order. Hi, I provide strong quality k2 spice papers and liquid for purchase at economical prices! Shipping is 100% concealed and delivery is secure.	Account of a seller commenting on their TikTok on how to place an order. Account of a seller commenting on their TikTok about the quality of their products, shipping, and delivery.	11 (2.0)

## Discussion

### Principal Findings

This observational infoveillance study analyzed 89 TikTok videos using the hashtag #k2spice and found that 40% of the content included discussing the use, solicitation to purchase or sell, or adverse effects of K2/Spice among prison populations. We also found a significant positive relationship between interactions with TikToks that included selling and buying activity among users. These exploratory results provide additional evidence that K2/Spice is a drug subject to abuse among the incarcerated population, and discussions about its use and sourcing are actively occurring on social media. Specifically, we found that the harmful effects of synthetic cannabinoid use are being recorded and shared on TikTok, and these TikToks are surfacing on K2/Spice-specific hashtag/keyword searches, potentially exposing other users to this content [24].

Our study specifically identified videos and comments that provided seller contact information and purported proof of product, indicating that content detailing where and how K2/Spice can be obtained in prison systems including through discrete packaging is being posted. We note that this appears to be a direct violation of TikTok drug content policies [25]. Several user-posted videos also demonstrated ways to evade drug screening procedures in prisons, such as placing K2/Spice on envelopes and greeting cards or putting it in a nasal spray, making it more difficult to detect the drug. This is consistent with previous findings noting the risks of its evasiveness and increasing use among this population due to being colorless, odorless, and highly potent [26]. Importantly, this open promotion of the sourcing of K2/Spice in state and federal correctional facilities may mean that screening procedures in prison facilities are being actively circumvented, introducing unique public health and substance abuse safety risks that require further study and response.

Our general findings align with several local news reports describing adverse psychological effects from K2/Spice use in state prisons, though the adverse effects of these synthetic drugs occur in all populations including those specifically involved in polysubstance abuse [27,28]. However, these consequences may be exacerbated in a population known to face social stigma, limited access to health care, unique psychological and mental health pressures, and risk of physical harm due to incarceration [5,26]. Additionally, continuing use of contraband substances while incarcerated may delay parole and the timely reintegration of individuals incarcerated for minor drug offenses because federal correctional facilities require the successful completion of rehabilitation programs [5].

Our results also suggest that higher user engagement on videos displaying K2-related adverse effects is associated with more comments intending to buy or sell K2/Spice. This may indicate that content specific to sourcing behavior may lead to higher interaction among TikTok users viewing behavioral-related content, warranting more targeted content moderation. This was observed despite TikTok guidelines stating that any content depicting or promoting drug consumption or solicitation will be removed, and violating accounts, when warranted, will be reported to legal authorities [25]. As TikTok is one of the newest and fastest growing social media platforms, it can be difficult to proactively identify content that violates user guidelines. However, regulation and the need to develop novel surveillance technologies is important as multiple studies have already identified unflagged calls for violence, harmful “internet challenges” (eg, Tide pod challenge), and antisemitism on the platform [12,29,30].

Synthetic cannabinoids are unregulated and often used in combination with other substances, which may increase the risk of adverse health effects [31]. In the presumed absence of rigorous prison screening standards for synthetic cannabinoids, specific harm may be brought on incarcerated populations who have recognized disparities in access to substance use treatment and psychosocial support [32]. Synthetic cannabinoids are increasingly easier to obtain and are deceptively described as cannabis cessation tools, emphasizing the importance of screening and peer-to-peer education [9]. The means by which synthetic cannabinoids such as K2/Spice are transported into prisons are also an issue of concern. Cards and letters are soaked with synthetic cannabinoids and serve as a substrate for substances such as K2/Spice, with varying concentrations creating “hot spots” that are unlikely to be known by incarcerated individuals [33].

There has been an increased pressure on social media companies to regulate the sale of illegal drugs occurring on their platforms as overdoses from opioid use, intentional and accidental self-injuries, and fentanyl poisonings continue to rise in the United States. Social media surveillance may represent a viable means to identify new trends and behaviors associated with this ongoing public health threat, particularly among traditionally hard to reach populations. The increasing popularity of synthetic cannabinoids has not been widely described in the literature, which has made it difficult to develop evidence-based interventions and policies to mitigate its harm. Results from this study can inform future research seeking to further characterize the use of social media platforms to promote both the use and trafficking of synthetic cannabinoids as well as lay the foundation for more targeted interventions in these uniquely vulnerable populations. Future studies should further validate exploratory results generated from this study, using additional hashtags and keywords, as well as data from other social media platforms, and augment these findings with other qualitative and quantitative research.

### Limitations

This study has certain limitations. Results are exploratory in nature, limited to a convenience sample of public content on TikTok, and may not be indicative of broader substance use patterns or themes in the general population. This study was also limited in its data collection and sample of data by the TikTok search algorithm, which displays videos based on their relevance to the keyword(s) searched at a particular point in time. Keywords associated with substance use are often monitored and removed by social media platforms, leading users to use pseudonyms or code words to evade content moderation. In fact, as of this writing, the term “k2spice” is now blocked as a search term on the TikTok platform, though using it in the context of a hashtag (#k2spice) during searches is not. Hence, the results of this study are not generalizable to all synthetic cannabinoid discussions or content occurring on the platform.

Although this study identified additional hashtags associated with synthetic cannabinoid use, it did not purposefully examine these hashtags for additional incarceration-related content and did not examine prison-specific hashtags (eg, #PrisonTikTok). Future studies should incorporate new, emerging, and trending substance-related and incarceration-related keywords, code words, and hashtags to better understand the changing social network dynamics of synthetic cannabinoid promotion and behavior. Moreover, as findings are based on a convenience sample generated by the platform’s own search algorithms, the scope of K2/Spice access and abuse among specific marginalized populations warrants additional investigation and inclusion of additional data sources that may be used by incarcerated populations. We were also unable to confirm if the contents described in this study were actually generated in correctional facilities due to the limited metadata available from TikTok accounts and posts.

### Conclusions

TikTok is a popular social media platform with millions of daily active users. Our study highlights the growing risk for the public and specific marginalized populations who may interact with and post psychoactive substance–related content on TikTok. Minimizing the potential individual and population-specific harm of this content on vulnerable prison populations should be a priority for platforms and the criminal justice system alike, as well as making attempts to strengthen substance abuse screening policies and evidence-based treatment to better ensure timely rehabilitation and reentry into society.

## Data Availability

The raw data supporting the conclusions of this article will be made available by the authors in a deidentified and aggregated format.
